# The Role of Advanced Glycation End-Product Levels Measured by Skin Autofluorescence in the Development of Mitral Annular Calcification

**DOI:** 10.3390/jcdd10090406

**Published:** 2023-09-20

**Authors:** Bedrettin Boyraz, Tezcan Peker

**Affiliations:** Cardiology Department, Medicalpark Hospital, Health Science Faculty, Mudanya University, Bursa 16950, Turkey; tezcan.peker@mudanya.edu.tr

**Keywords:** advanced glycation end-products, mitral annular calcification, skin autofluorescence

## Abstract

As a person ages, mitral annular calcification develops in the mitral annulus with increasing frequency. Lipid deposition, inflammation, and aging-related degeneration have been cited as potential causes of this pathophysiology, though there is currently no conclusive evidence to support this. AGEs accumulate in tissues due to the glycation of proteins and lipids, increasing the release of proinflammatory cytokines secondary to oxidative stress through the AGE receptor. The AGE levels increase in diabetic microvascular complications and degenerative aortic valve disease. Our study was planned prospectively as a case–control study involving 94 MAC-positive patients and 94 MAC-negative patients. The demographics, echocardiographic data and AGE levels of the patients were measured and recorded using the skin autofluorescence method. AGE levels were significantly higher in the MAC-positive patient group (3.2 vs. 2.7; *p* < 0.001). The AGE levels were observed as an independent predictor of MAC development in a regression analysis (OR: 8.05, 95% CI: 3.74–17.33, *p* < 0.001). In a ROC-curve analysis, the AUC was 0.79 (95% CI: 0.72–0.85). At a cut-off value of 2.7, 79.7% sensitivity and 69.1% specificity were observed. AGE levels can be used to cheaply, easily and non-invasively identify patients at risk of developing MAC.

## 1. Introduction

The mitral valve annulus (MVA) is the fibrous skeletal tissue that forms the border between the left atrium (LA) and the left ventricle (LV), to which the anterior (AML) and posterior mitral leaflets (PML) are attached. Mitral annular calcification (MAC) is defined as calcification along the MVA, often extending to the PML and less frequently to the AML, and it is secondary to aging-related degeneration, lipid accumulation, and inflammation in the MVA [1,2]. Histological examinations revealed a calcified collagen matrix, necrotic tissue, angiogenesis tissue and inflammatory cell infiltration in the MVA [3,4]. Mitral regurgitation (MR), mitral valve stenosis (MS), conduction disorders and left ventricular outflow tract obstruction may result from damage to the mitral valve (MV) [5]. The development of MAC leads to increased cardiovascular mortality and morbidity, as well as a greater likelihood of coronary artery disease (CAD), heart failure (HF) and atrial fibrillation (AF) [6,7]. MAC has been associated with C-reactive protein (CRP) and interleukin-6 (IL-6) [8]. Its incidence increases with age (10–15% at age 70) and it is more prevalent in females [3] as well as in people with endothelial dysfunction, atherosclerosis, peripheral arterial disease, chronic renal failure (CRI) and diabetes mellitus (DM) [5,6,7]. It also increases cardiovascular mortality and morbidity, both due to the disease itself and to the increase in operational risks in patients that require cardiac interventions for other reasons [9]. However, its pathophysiology has yet to be fully determined. Tissue degeneration due to aging has been blamed, but MAC does not occur in all elderly people [10]. For this reason, research and opinions about pathophysiology are based on conditions that increase risk factors, the most important of these being aging. Aging causes deterioration at the cellular and tissue level. As a result, fibrosis, inflammatory cell accumulation and tissue calcification occur. Some researchers have suggested that impaired calcium and phosphate metabolism may affect the condition due to female gender or CRI. These considerations highlight the deficiencies in our understanding of its pathophysiology [11].

Non-enzymatic reactions involving the terminal amino groups of amino acids or the side-chain amino groups of lysine and arginine within proteins, along with carbonyl groups from reducing sugars, lead to the formation of Schiff bases. Under increased oxidative stress conditions, these Schiff bases undergo further glycoxidation reactions, ultimately producing highly reactive 1,2-dicarbonyl compounds. These 1,2-dicarbonyl compounds interact with the amino groups within proteins, leading to the creation of crosslinks both within and between proteins. The intricate series of reactions responsible for the formation of advanced glycation end-products (AGEs) are collectively known as Maillard reactions—chemical processes that occur between the carbonyl groups found in reducing sugars and the N-terminal amine groups found in amino acids, peptides or proteins [12]. Because of these reactions, the normal physiological functions of proteins that are crosslinked by AGEs, including receptor proteins and enzymes, are inactivated. These modifications to proteins and the small molecules produced as a result of their breakdown are collectively referred to as advanced glycation end-products [13,14]. AGEs resulting from non-enzymatic glycation and oxidation of proteins and lipids cause endothelial dysfunction and impaired renal function due to their accumulation in tissues, type 1–4 collagen and on the cellular level. By inducing oxidative stress, AGEs trigger the activation of various stress-responsive transcription factors, and inflammatory molecules such as cytokines and acute-phase proteins (e.g., NFκB, IL-1β, TNF-α, IL-6) are generated through the receptor pathway as a result [15,16,17]. Increased exogenous uptake occurs due to metabolic syndrome, obesity, fast-food consumption, and Western dietary habits, while increased endogenous production occurs due to issues such as oxidative stress, chronic inflammation, hyperlipidemia and DM. AGE accumulation increases with aging and in cases in which clearance decreases, such as in CRI [18]. In addition to measuring the serum level, the level can be measured non-invasively with the skin autofluorescence method, since it accumulates in the skin and can be used to predict total AGE accumulation [19]. Studies have shown that AGEs play a role in the development of microvascular complications of DM, diabetic heart failure, endothelial dysfunction and vascular stiffness development by causing calcium hemostasis disorder after interaction with cardiac ryanodine and SERCA-2a; this can be used to predict cardiovascular mortality in type 2 DM patients [20,21,22].

These data support the hypothesis that AGEs may play a role in the pathophysiology of MAC. Our study investigates the relationship between the amount of AGEs measured using the skin autofluorescence method and MAC.

## 2. Materials and Methods

Our study was planned and conducted as a prospective case–control study in accordance with the 1967 Helsinki Declaration. We obtained informed consent from all patients included in the study with the approval of the human research ethics committee of Istinye University (meeting 2023/07, protocol 23/218). Our study included 94 patients who were screened for MAC. The participants had no history of previous valve or cardiac surgery, no previous invasive intervention to the mitral valve, no history of rheumatic valve disease, no active infection, no end-stage renal failure and no liver failure. People with similar demographic characteristics were included as the control group. The exclusion criteria are summarized in Table 1.

The patient group involved people who had previously been diagnosed with mitral annular calcification and received regular follow-up at our hospital. Demographic characteristics and echocardiographic findings of the patients and control group were obtained from their anamnesis and patient files and recorded, as well as the disease duration of patients diagnosed with DM and the last level of their HbA1c tests. Inter-group comparison of the diabetic patients was also performed.

The skin autofluorescence measurements of the participants were the average of 3 measurements made by an individual who did not know which group the subjects belonged to. The AGE reader™ (DiagnOptics Technologies B.V., Groningen, The Netherlands) was used to obtain the measurements, which were taken from the inner surface of the forearm of the patients according to the manufacturer’s instructions. No measurements were taken from areas with scars or pigmentation disorders.

In statistical analysis, while analyzing the normality and variance of the parameters, the Kolmogorov–Smirnov test and Shapiro–Wilk tests were used. The chi-square test and Fisher’s exact test were used to analyze the categorical variables, categorical variables were expressed in numbers and percentages. After that, the Mann–Whitney U test or Student’s *t*-test was applied to the numerical variables, and the data were summarized as median and 25–75% interquartile range (IQR) or mean ± standard deviation. In the regression analysis, univariate regression analysis was performed with all parameters, and multivariate regression analysis was performed using parameters with a *p*-value < 0.1. Receiver operating characteristic curve (ROC-curve) analysis was performed to evaluate AGE levels and perform MAC prediction, then the cut-off value, sensitivity and specificity were given, and the data were summarized with a 95% confidence interval (CI). A *p*-value < 0.05 was accepted as the significance level. SPSS 23 (IBM Corp. Released 2015. IBM SPSS Statistics for Windows, Version 23.0. Armonk, NY, USA: IBM Corp.) and STATA.17 (Stata Corp. 2021. Stata Statistical Software: Release 17. College Station, TX, USA: Stata Corp LLC.) were used for the statistical analysis.

## 3. Results

Our study included 94 MAC-positive patients and 94 MAC-negative patients. Female gender dominance was observed in the MAC-positive group, while male gender dominance was observed in the other group. More than two-thirds of the patients in both groups exhibited signs of hypertension (HT). The statistically significant HT frequency was observed to be higher in the group with MAC. The mean age of both groups was 70 and there was no statistical significance between them. More than half of the patients in both groups had CAD. More than one-third of the patients in both groups were smokers. Patients with MAC have a more frequent history of stroke. The frequency of chronic obstructive pulmonary disease (COPD) was higher in the MAC-positive group. Although the frequency of AF was numerically higher in the MAC-positive group, it failed to reach statistical significance. Approximately half of the patients in both groups had DM; however, no significant difference was observed between the groups in terms of DM frequency and the duration of DM. The hemoglobin A1c (HbA1c) levels of the patients were examined, and the HbA1c levels were significantly higher in the MAC-positive group. The body mass index (BMI) was similar in both groups. The echocardiography data were examined, and the patients’ ejection fractions (EF) were similar. The number of patients with MR > 1 degree was significantly higher in the group with MAC positivity and LA diameter; like MR, it was higher in the MAC-positive group. AGE levels were significantly higher in the MAC positive group. The data are summarized in Table 2.

**Figure 1:**
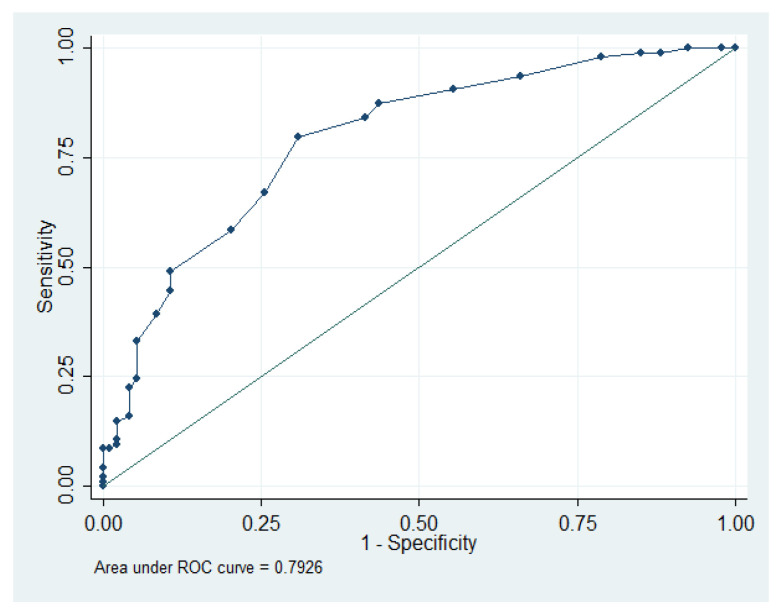
AGE levels that predict MAC positivity.

We used ROC-curve analysis to test the predictive power of AGE levels with regard to MAC positivity. The area under the curve (AUC) was 0.792; 95% CI: 0.728–0.856. Further analysis revealed a sensitivity of 79.7% and specificity of 69.1% at the cut-off value of 2.7 (Figure 1).

Multivariate regression analysis was performed based on the following parameters: age, female gender, HT, stroke, COPD, HbA1c levels, AGEs, LA diameter and MR > 1, which, in the univariate regression analysis, resulted in a *p*-value less than 0.1. The analysis identified LA diameter, AGEs and stroke as independent predictors of MAC. The results of this analysis are summarized in Table 3.

When DM patients were excluded from the analysis, a statistical analysis was performed. The MAC-positive group included 48 patients with DM, while in the MAC-negative group, 47 patients did not have DM. In this analysis, the AGEs values of MAC-positive patients had a median of 2.8 (2.6–3.2 IQR), while in the MAC-negative group, the median was 2.2 (1.8–2.4 IQR), with a *p*-value < 0.001. Additionally, in MAC-positive patients without DM, the AGEs values were significantly higher compared to those of MAC-negative patients. There were 55 patients with AGEs of three and above: 9 of them in the control group and 46 in the patient group. The creatinine values of these patients were mean 1.03 ± 0.26.

## 4. Discussion

Our results show that AGE levels are independent predictors of MAC, with 79% sensitivity and 69% specificity in patients with a cut-off value above 2.7. These findings are indicative of a relationship between AGE levels and MAC. Although we cannot be certain that AGEs accumulate in the MVA, since we did not perform a histological study at the tissue level, it can be concluded that the risk of MAC increases in people with a high accumulation of AGEs in the skin. This demonstrates that non-invasive measurement of AGEs in a very short time in at-risk demographics will enable timely detection of people at a high risk of MAC, allowing further diagnostic and treatment methods to be implemented at an early stage. The presence of inflammatory cell accumulation, calcification, and fibrosis in the valve in the pathophysiology of the development of MAC supports the role of AGEs. The accumulation of AGEs may be the result of increased exogenous uptake, endogenous production and decreased clearance. There was a notable increase in the amount of AGEs in patients who ate a Western diet [23]. As mentioned previously, the glycation of proteins, lipids and nucleic acids increased due to the increase in high glucose, lipid and oxidative stress. These structurally damaged substances accumulate at the tissue level and disrupt the functions and architecture of the structure. As a result, proinflammatory cells and cytokines are released and tissue inflammation occurs. Here, a DM-induced increase in the amount of AGEs may contribute to the development of MAC due to hyperglycemia, dyslipidemia lipid elevation and HT endothelial dysfunction. The presence not only of calcification but also inflammation at the tissue level in histological studies performed on tissue that develops MAC suggests that degeneration does not exclusively occur in the pathophysiology, but also an additional pathway that increases inflammation. Among the underlying reasons for the AUC value to reach 0.79 levels in terms of predicting the development of MAC is the potential role of AGEs in the pathophysiology. Both the accumulation in type 1–4 collagen tissue and the possibility of accumulation at the MVA level may cause defects in both valve structure and functions and MVA architecture. Histological studies on this condition may clarify the issue. Another reason may be that AGE levels are elevated in the vast majority of conditions that are risk factors for the development of MAC. If there is a corresponding increase, then it will provide us with a good, non-invasive, easy-to-apply, low-cost test with which to determine the risk of MAC development.

Predictors of MAC include advanced age, female gender, end-stage renal disease requiring hemodialysis treatment, high aortic and coronary calcium load, obesity, dyslipidemia and DM. Our study found that LA diameter and stroke were predictors in addition to AGE levels. Our univariate logistic regression analysis identified age, female gender, HT, stroke, COPD, HbA1c levels, AGEs, MR > 1 and LA diameter as predictors of MAC. We believe that the reason for the loss of predictive power in multivariate analysis is the advanced age of our patient groups (mean > 70). In general, we believe that age loses its predictive power in the multivariate analysis due to the inclusion of elderly patients in the study and the case–control study design. We conclude that factors such as obesity, dyslipidemia, DM, age, female gender and HT lose their predictive power in the multivariate analysis due to the presence of such high rates of HT, DM and dyslipidemia patients in the study. We believed that the case–control study design would be the most accurate, since the main purpose of our study was to test the predictive power of AGE levels with regard to MAC. Since LA diameter and stroke are factors that may be caused by MAC, their predictive power has been observed in these patients.

When the differences between both groups were observed, it was noted that female gender was significantly more prevalent in the MAC-positive group. This supports the literature, which states that female gender is an independent predictor for MAC. Male gender, on the other hand, is a risk factor for atherosclerosis, which may cause confusion in terms of pathophysiological female gender being a risk factor for MAC. However, abnormal calcium phosphate production and post-menopausal osteoporosis are often blamed for this [24].

As stated by the literature, the presence of HT was higher (greater than 80%) in the MAC-positive group, though it was observed in over 70% of patients in the MAC-negative group. AGEs are catabolized from proximal renal tubules, and apoptosis and inflammation occur in renal proximal cells due to the increase in AGEs. This leads to a subsequent increase in the reabsorption of sodium, which contributes to the development of HT, and increases in AGE levels contribute to the development of HT and, therefore, MAC [25].

The frequency of DM was around 50% in both groups, and the duration of DM was 10 years in both groups. DM is a risk factor for the development of MAC; it is mainly accused of increasing the development of atherosclerosis. Increases in AGE levels are observed in DM patients. The glycation of proteins, nucleic acids and lipids, secondary to a hyperglycemic state or inflammation, plays a fundamental role in the formation of AGEs. Accordingly, these substances begin to accumulate in the tissues, resulting in tissue dysfunction. This leads to an increase in the level of microvascular complications of DM. Rezaei, M. et al. support this assumption; their studies show that AGEs measurements are valuable in the follow-up of DM complications, but they were unable to prove a relationship between HbA1c levels and blood sugar regulation and AGE levels in their work [26]. One reason for the development of MAC may be the accumulation of AGEs in MVA; to confirm this, further tissue analyses are required. The lack of difference between the groups in terms of DM frequency does not necessarily indicate that there is no difference in blood sugar regulation. Therefore, the HbA1c levels of patients with DM were compared. HbA1c levels of 7.35 were observed in the MAC-positive group, while they were significantly lower than 7.0 in the other group. This confirmed that blood glucose regulation was worse in the MAC-positive group, at least in the last 3 months.

Obesity and hyperlipidemia are risk factors for both CAD and MAC. Obesity contributes to this effect by increasing the frequency of diseases such as hyperlipidemia, HT and DM due to metabolic syndrome. Hyperlipidemia increases lipid deposition in the tissues and thus accelerates plaque development; as a result, the plaque degenerates into an anoxic condition due to malnutrition in the plaque, and ultimately, calcification occurs. MAC development is mainly observed secondary to lipid accumulation and calcification. Hyperlipidemia increases both lipid accumulation and calcification accumulation by creating inflammation and anoxic condition in the tissue [27]. This also leads to an increase in the production and accumulation of AGEs in the existing tissue, which contributes to the current situation. Sanchis et al. and Deng et al. also blamed this mechanism in their studies, which showcased the relationship between aortic valve calcification and serum AGE levels [28,29]. We also believe that the increase in AGE levels in our study affects the development of MAC by using similar mechanisms.

COPD was identified as a risk factor for MAC in univariate analysis, and its frequency was significantly higher in the MAC-positive group. There was no significant difference between the groups in terms of smoking, which is a related condition. We believe that the main effect of smoking and COPD on the development of MAC is that it creates anoxic conditions in the body and increases intra-plaque bleeding and related inflammation. We suggest that as a result of the existing anoxic environment and inflammation, the production of AGEs increases, and their accumulation increases in tissues, especially in cardiovascular structures. Smoking is a risk factor for the development of atherosclerosis and MAC [30]. The most basic risk factor for the development of COPD is smoking, but not every smoker develops COPD. This shows that the effect of smoking is greater in patients with COPD. Naturally, it would be reasonable to predict that COPD development would have a higher predictive power for MAC than smoking.

MAC can lead to increased incidence of HF, MR or MS, AF and stroke. Calcification of the MV cusps due to MAC can cause adhesions and coaptation defects. While adhesions more frequently increase MS, MR also increases as a result of coaptation defects. In one study, 11.7% of 1494 MAC-positive people also had MR; 0.05% had MS; and 8.2% of these people had LA dilatation. [31]. Our study detected MR > 1 degree in 30.9% of the MAC-positive patients. Depending on the increase in MR, volume and pressure overload occur in the LA and LV. This situation paves the way for dilatation in LA, and an associated increase in the incidence of AF development in the MAC-positive group, which supports the current literature findings. The association of MAC with AF was demonstrated in the Framingham Heart study, and in another study by Wesley et al., who concluded that the presence of MAC is an independent predictor of the risk of developing AF [32,33]. Stroke may develop secondary to AF in patients with MAC or secondary to valve calcification. Although the frequency of AF was numerically high, no significant difference was observed. In addition, stroke was observed as an independent risk factor for MAC in the regression analysis. LA diameter and MR > 1 degree are interrelated conditions. Secondary to the existing MR, the LA remains under pressure and volume overload and, subsequently, LA dilatation occurs. The incidence of MR > 1 degree and LA diameter was significantly higher in the group with MAC.

In the studies by Nakamura et al., it was demonstrated that the molecule telmisartan reduces soluble advanced glycation end-product receptor (RAGE) levels. Meanwhile, in their research, Matsui et al. showed that the molecule irbesartan decreased AGE-related tubular cell injury. In their study, Ono et al. indicated that the molecule candesartan suppressed AGE levels in individuals with type 2 diabetes and essential hypertension. Additionally, Sebekova et al. suggested that the molecule ramipril potentially improved parameters in non-diabetic nephropathy patients, possibly acting through AGEs. Alagebrium (ALT-711) is known as an AGE-breaker and can reduce AGE levels. Studies have shown conflicting results, with some indicating a reduction in total arterial compliance, a decrease in left ventricular mass, and an improvement in arterial endothelial function, while others have found no significant effect, leading to some confusion in this regard [34,35,36,37,38,39].

## 5. Conclusions

AGE levels are an independent predictor for MAC, which can predict MAC at the level of 2.7 with 79% sensitivity and 69% specificity. AGE levels can be measured easily, non-invasively and at minimal cost with the AGE-reader device, revealing people at greater risk of MAC development.

## Figures and Tables

**Table 1:** Exclusion criteria.

- History of previous valve or cardiac surgery or intervention. - History of rheumatic valve disease. - Active infection or malignancies. - End-stage renal disease. - Liver failure. - History of infective endocarditis.

**Table 2:** Demographic, echocardiographic and laboratory data.

Variable	MAC-Positive	MAC-Negative	*p* Value
Number of patients	94	94	
Gender (female)	55 (58.5%)	40 (42.6%)	0.02
DM	46 (48.9%)	47 (50%)	0.5
Hypertension	78 (83%)	67 (71.3%)	0.041
Hyperlipidemia	57 (60.6%)	59 (62.8%)	0.4
Smoking	33 (35.1%)	35 (37.2%)	0.4
Coronary artery disease	54 (57.4%)	52 (55.3%)	0.4
Stroke	11 (11.7%)	3 (3.2%)	0.02
COPD	21 (22.3%)	9 (9.6%)	0.01
Atrial fibrillation	14 (14.9%)	8 (8.5%)	0.1
MR > 1 degree	29 (30.9%)	9 (9.6%)	<0.001
Age	72.04 ± 7.26	70.04 ± 6.22	0.07
BMI	29.27 (26.55–31.97)	28.9 (27.6–32.8)	0.4
DM year	10 (7–15)	10 (6–15)	0.8
HbA1c	7.35 (6.7–8.21)	7 (6.7–7.3)	0.03
AGEs	3.2 (2.8–3.7)	2.7 (2.4–2.9)	<0.001
Ejection fraction	55 (48.75–60)	55 (50–60)	0.18
Left atrium diameter (cm)	4.15 (3.87–4.5)	3.8 (3.5–4.1)	<0.001
Triglyceride	120 (98–155 IQR)	113 (93–142 IQR)	0.1
Low-density lipoprotein	116.6 (89–133)	94.5 (68–114.25)	0.01
Creatin	0.95 (0.78–1.12)	0.90 (0.78–1.04)	0.4

Abbreviations: AGEs: Advanced glycation end-products; BMI: body mass index; cm: centimeters; COPD: chronic obstructive pulmonary disease; DM: diabetes mellitus; MAC: mitral annular calcification; MR: mitral regurgitation.

**Table 3:** Regression analysis for predictors of MAC.

	Univariate Regression Analysis			Multivariate Regression Analysis		
Variable	OR	95% CI	*p* Value	OR	95% CI	*p* Value
DM year	1.19	0.93–1.04	0.6			
Age	1.03	0.99–1.07	0.074	0.99	0.88–1.11	0.8
Gender (Female)	1.90	1.06–3.39	0.029	2.22	0.64–7.61	0.2
DM	0.95	0.54–1.69	0.8	-	-	-
BMI	1.03	0.96–1.11	0.2	-	-	-
Hypertension	1.96	0.97–3.95	0.058	0.28	0.03–2.06	0.2
Hyperlipidemia	0.91	0.50–1.64	0.7	-	-	-
Smoking	0.91	0.50–1.65	0.7	-	-	-
CAD	1.09	0.61–1.94	0.7	-	-	-
Stroke	4.02	1.08–14.91	0.03	5.55	1.27–24.25	0.023
COPD	2.71	1.17–6.30	0.02	7.00	0.89–54.90	0.064
AF	1.88	0.74–4.72	0.178	-	-	-
HbA1c	1.89	1.13–3.15	0.01	1.40	0.63–3.13	0.4
AGEs	9.00	4.25–19.05	<0.001	8.05	3.74–17.33	<0.001
EF	0.96	0.93–1.00	0.107	-	-	-
LA	5.07	2.25–11.42	<0.001	3.76	1.47–9.64	0.006
MR > 1 degree	4.21	1.86–9.51	0.001	4.07	0.95–17.47	0.059

Abbreviations: AF: atrial fibrillation; AGEs: advanced glycation end-products; BMI: body mass index; CAD: coronary artery disease; COPD: chronic obstructive pulmonary disease; DM: diabetes mellitus; EF: ejection fraction; LA: left atrium; MR: mitral regurgitation; OR: odds ratio.

## Data Availability

The data can be made available upon reasonable request through a data access committee, institutional review board or the authors themselves.

## Abbreviations

AGE	Advanced glycation end-product.
AF	Atrial fibrillation.
AML	Anterior mitral leaflet.
AUC	Area under the curve.
BMI	Body mass index.
CAD	Coronary artery disease.
CI	Confidence interval.
COPD	Chronic obstructive pulmonary disease.
CRI	Chronic renal insufficiency.
CRP	C-reactive protein.
DM	Diabetes Mellitus.
EF	Ejection fraction.
HF	Heart failure.
HT	Hypertension.
IL-6	Interleukin 6.
IQR	Inter quartile range.
LA	Left atrium.
LV	Left ventricle.
MAC	Mitral annular calcification.
MR	Mitral regurgitation.
MS	Mitral stenosis.
MV	Mitral valve.
MVA	Mitral valve annulus.
OR	Odds ratio.
PML	Posterior mitral leaflet.
ROC	Receiver operating characteristics.
RAGE	Receptor of AGE.
